# A Critical Evaluation of Methodological Discrepancies in Quantifying Steel Slag CO_2_ Sequestration

**DOI:** 10.3390/ma19194032

**Published:** 2026-09-22

**Authors:** Mingming Wang, Chunyao Song, Zhonglun Zhang, Xiao Chen, Chengying Bai, Zhilong Zheng, Xiaofan Cai, Zihan Feng, Shengnan Zhou, Suihua Guo

**Affiliations:** 1 School of Materials Science and Engineering, Wuhan University of Technology, Wuhan 430070, Chinazzl1111@whut.edu.cn (Z.Z.); 2 China Building Materials Academy Co., Ltd., Beijing 100024, Chinash_guo@sina.com (S.G.); 3 College of Materials Science and Chemical Engineering, Harbin Engineering University, Harbin 150001, China

**Keywords:** steel slag, steel slag carbonation, CO_2_ sequestration, CO_2_ sequestration capacity, solid waste utilization

## Abstract

Steel slag, rich in free CaO/MgO, calcium silicates, and calcium ferrites, suits engineering, boosting alkaline solid waste and carbonating CO_2_ minerals. However, studies interpret CO_2_ sequestration capacity differently. Metrics like CO_2_ sequestration capacity, mass gain, and carbonate content are lumped as capture measures, blocking cross-study comparison and inconsistent carbonation assessments. The study rigorously evaluates seven conventional measurement techniques: thermogravimetric analysis, mass gain measurement, gas consumption monitoring, acid digestion method, elemental analysis, combustion-infrared absorption spectrometry, and X-ray diffraction, thoroughly discussing their quantifiable targets, relevant scopes, systematic biases, and failure scenarios. Representative data show 7-day CO_2_ sequestration values of 6.5–10.2 g CO_2_/100 g initial sample across four analytical routes; stepwise-TG, tangential-TG, combustion-infrared AD-TIC, and acid-digestion HT-TIC gave 9.0, 6.5, 10.2, and 9.7 g CO_2_/100 g, respectively, corresponding to a highest-to-lowest spread of 3.7 g CO_2_/100 g (36.3% of the highest value). Analytical findings indicate that variances among testing techniques arise not only from arbitrary measurement errors but mainly from differences in specific carbon pools, protocols for sample pre-treatment, criteria for identifying carbonate species, categorization of gas–liquid–solid phase limits, and methods for assessing statistical uncertainty. Consequently, this document suggests a quadruple-tiered evidence chain structure, encompassing the material background, process mass equilibrium, product-associated carbon measurement, and statistical fusion layers. An array of functional standards has been formulated, encompassing the removal of background baselines, confirmation of mass closure, four-point cross-validation, and broadened reporting of uncertainties. The suggested model converts the measurement of steel slag’s carbon capture ability from isolated, singular measurements to a system that is traceable, confirmable, and verifiable with multiple evidence sources. This offers strong methodological backing for large-scale lab experiments, expanding processes, confirming carbon emission reductions, and developing uniform testing procedures. To improve operational clarity, the framework explicitly distinguishes background solid inorganic carbon, newly mineralized stable solid carbon, liquid-phase dissolved inorganic carbon, physically retained CO_2_, and crystalline carbonate carbon; it also introduces quantitative decision bands for mass closure and cross-method agreement and demonstrates reconciliation using a published multi-method BOF steel-slag dataset.

## 1. Introduction

Industrial solid waste (ISW)—from metallurgical, coal, mining, and chemical sectors—poses environmental threats yet offers resource potential [1,2,3]. High-value routes include metal recovery [4,5,6], construction materials (supplementary cementitious materials and precursors) [7,8,9], functional adsorbents/catalysts [10,11], porous materials [12,13], energy production/storage [14], and others [15,16,17]. Despite emerging technologies, challenges like feedstock variability, cost, and secondary pollution remain. Transforming CO_2_ into carbonates that are thermodynamically stable through alkaline solid wastes presents a hopeful technical path combining solid waste valorization with decreased carbon emissions [18,19,20]. Owing to its plentiful raw materials, durable mineral products, and minimal risk of carbon leakage, this mineral carbonation technique is acknowledged as a key strategy for the combined progress of industrial solid waste recycling and carbon reduction [21,22,23,24,25]. The iron and steel sector contributes significantly to worldwide human-made carbon emissions, with steel slag being the largest alkaline solid by-product produced in steel production. Figure 1 displays the yearly statistical figures for steel slag production in key countries and regions globally [26]. The yearly production of steel slag in China ranges from 150 to 200 million tons, yet its overall usage rate remains below 40% [27], significantly below the 70% rate seen in advanced nations like Europe, the United States, and Japan [28]. The presence of carbonation-active elements in steel slag, such as unbound calcium oxide, magnesium oxide, and calcium silicates, bestows upon this solid waste a high potential for CO_2_ mineral carbonation [27,29,30].

Steel slag carbonation technology can not only achieve permanent CO_2_ sequestration, but also eliminate volume expansion instability caused by residual f-CaO and f-MgO compared to traditional landfill disposal and low-value utilization (Figure 2 compares mainstream steel slag utilization strategies in typical countries and regions [31]). As a result, this technology enables the high-value reuse of steel slag in building materials, road works, and solid waste cementitious materials [27,28,32,33,34,35]. However, the key bottleneck limiting the industrialization of laboratory-scale steel slag carbonation research is the accurate quantification of the actual CO_2_ sequestration of steel slag samples, that is, the determination of CO_2_ sequestration capacity [36,37].

To date, there is no uniform quantitative standard for CO_2_ sequestration capacity quantification in academia and industry. Existing research shows substantial divergences in the definition and statistical caliber of CO_2_ sequestration capacity. In particular, relevant indicators vary widely across the literature: some studies have used CO_2_ sequestration capacity per unit mass of coarse steel slag, while others have selected newly produced carbonates in carbonated products for CO_2_ absorption. Other commonly used indicators include the carbonation conversion rate, the theoretical CO_2_ sequestration potential utilization ratio, and the gaseous CO_2_ utilization efficiency. In addition, in numerous studies, the consumption of gaseous CO_2_, mass gain, and crystalline carbonate content are directly considered to be equivalent to CO_2_ sequestration capacity [38,39,40,41]. In the absence of unified quantification boundaries, these homonymous indicators correspond to completely different carbon pools. This inconsistency leads to poor comparability between results across studies and even to significant bias in CO_2_ sequestration data from the same slag samples when tested by different methods. Kurihara et al. [42] studied the effects of seven mainstream detection methods on the quantification of CO_2_ sequestration and showed a systematic bias of more than 30% for different detection methods.

At present, domestic and international research on steel slag carbonation focuses on carbonation reaction mechanisms, external influencing factors, and engineering application performance [27,43,44,45,46,47]. Targeted critical evaluation of the inherent non-comparability and systemic structural bias in CO_2_ sequestration quantification is still lacking.

Accordingly, this paper focuses on methodologies for quantifying CO_2_ sequestration capacity as the main research thread. It first briefly summarizes the characteristics of steel slag carbonation reactions and their effects on quantitative measurement. Subsequently, seven mainstream quantification approaches are evaluated from the perspectives of measurable targets and applicable scopes, followed by an in-depth analysis of the structural origins of discrepancies among these methods. Furthermore, an operational four-layer evidence chain framework is proposed in this work. Rather than pursuing a single universal testing method, this study aims to establish a transparent, reproducible, and auditable collaborative quantification paradigm. The proposed paradigm enables consistent comparability of steel slag CO_2_ sequestration data acquired at various scales, across diverse carbonation processes, and for distinct research objectives.

## 2. Premise for Quantifying the CO_2_ Sequestration Capacity of Steel Slag: Reaction Characteristics and Interference from Key Factors

The premise for quantifying steel slag CO_2_ sequestration capacity is to clarify the fundamental mechanisms and influencing factors of steel slag mineral carbonation. Variations in carbonation pathways and inherent slag properties directly determine the selection of quantification approaches and the accuracy of the resulting CO_2_ sequestration data. This chapter systematically explains the basic principles, reaction pathways, and dominant influencing factors of steel slag carbonation, laying a solid foundation for the subsequent classification and critical evaluation of quantification methodologies.

The essence of steel slag carbonation reactions relies on reactive Ca- and Mg-bearing mineral phases in steel slag, including f-CaO, f-MgO, Ca(OH)_2_, C_2_S, and C_2_S. These components undergo carbonation reactions with CO_2_ to generate thermodynamically stable carbonate-dominated inorganic carbon phases, thereby realizing permanent CO_2_ sequestration within the steel slag matrix [26,48,49]. Two distinct carbonation pathways have been identified: direct carbonation and indirect carbonation. For direct carbonation, gaseous CO_2_ dissolves in water films or pore solutions to produce HCO_3_^−^/CO_3_^2−^, which subsequently precipitates with leached Ca^2+^/Mg^2+^. Indirect carbonation involves the leaching of metallic cations via acidic or complexing media, followed by carbonate precipitation [27,28]. Accordingly, the CO_2_ sequestration capacity of steel slag is not solely governed by CO_2_ supply, but synergistically controlled by leachable Ca/Mg content, aqueous chemical environment, diffusion pathways, and microstructure of product layers [50,51,52].

From a kinetic perspective, steel slag carbonation generally proceeds through three sequential stages: rapid surface reaction at the initial stage, diffusion-controlled reaction across product layers in the intermediate stage, and passivation at the late stage [53,54,55,56]. Such multi-stage characteristics fundamentally compromise the reliability of diverse quantification methods. The gas consumption method sensitively captures early-stage CO_2_ uptake rates, yet it tends to falsely count temporarily stored dissolved inorganic carbon (DIC) in liquid phases or entrapped pore CO_2_ as mineralized solid carbon. The mass gain method characterizes the overall mass variation in molded specimens, whereas its results are susceptible to interferences from hydration, moisture absorption, residual salts, and fine particle loss. Thermogravimetric analysis (TGA), acid digestion, and elemental analysis target stable inorganic carbon in solid carbonated products, yet challenges remain regarding sample representativeness, consistent pretreatment protocols, and effective separation of distinct carbon speciation.

The inherent heterogeneity of steel slag samples constitutes a fundamental source of uncertainty universal to all quantification techniques. Particle size fractions, cooling regimes during steelmaking, and aging degrees induce a heterogeneous distribution of free CaO, C_2_S RO phases, glassy phases, and native carbonates within the slag matrix. Substantial discrepancies exist in phase assemblages across testing scales: milligram-scale specimens for TGA, gram-scale samples for acid digestion, and kilogram-scale structural components exhibit inconsistent mineralogical compositions. To avoid confusing sampling bias with method-induced deviations, the oxide composition, mineral phases, particle size distribution, moisture content, loss on ignition, and total inorganic carbon background of raw steel slag should be fully documented prior to CO_2_ sequestration quantification, alongside adequate homogenization and multi-point sampling strategies.

Process parameters exert profound impacts on the defined boundaries of CO_2_ sequestration quantification. Elevated CO_2_ partial pressure generally enhances mass transfer driving force; nevertheless, the marginal improvement diminishes once cation leaching or product-layer diffusion becomes the rate-limiting step. An excessively low liquid-solid ratio restricts ionic migration, while an overly high ratio dilutes reactive mineral phases, elevates temporary liquid-phase DIC storage, and increases energy consumption during post-processing. Increasing temperature accelerates reaction kinetics and metallic cation leaching, yet simultaneously reduces aqueous CO_2_ solubility and potentially alters carbonate crystal polymorphs or the stability of hydration products. Consequently, all reported CO_2_ sequestration values must be accompanied by full documentation of reaction regimes, sample conditions, and well-defined quantification boundaries.

In summary, the core conclusion of this chapter, supporting subsequent method evaluation, is that steel slag CO_2_ sequestration quantification corresponds to a multi-carbon-pool system involving coupled gas, liquid, solid, and crystalline phases, rather than a single calcium carbonate formation reaction. Hydration by-products interfere with thermal decomposition intervals in TGA measurements, pore water and residual salts disrupt mass gain testing results, liquid-phase DIC renders recorded gaseous CO_2_ consumption higher than newly formed solid carbon, amorphous carbonates lead to underestimated carbon content via XRD quantification, and unsubtracted native carbonates in raw slag introduce systematic positive biases for all solid-phase quantification methods. All aforementioned interference factors shall be explicitly stated during method selection and standardized data reporting in subsequent sections.

## 3. Critical Evaluation of Mainstream Quantification Approaches for the CO_2_ Sequestration Capacity of Steel Slag

The core objective of quantifying steel slag CO_2_ sequestration capacity lies in the accurate determination of carbonate contents generated after carbonation reactions of steel slag. Based on distinct measurement dimensions, existing quantification techniques are categorized into three groups: gas-phase methods (gas consumption method), solid-phase methods (mass gain method, TGA, acid digestion method, elemental analysis, and combustion-infrared absorption spectrometry), and crystalline-phase methods (XRD). Figure 3 schematically illustrates these mainstream quantification approaches for steel slag CO_2_ sequestration. Table 1 compares the characteristics of different quantitative characterization methods for CO_2_ sequestration via mineral carbonation. The gas consumption method quantifies the total CO_2_ consumed throughout the reaction process; TGA, acid digestion, elemental analysis, and combustion-infrared absorption spectrometry measure the total carbonate or inorganic carbon retained within solid products; XRD exclusively quantifies crystalline carbonate phases contained in specimens. Nevertheless, the physical quantities characterized by these techniques are not interchangeable, and method selection must be tailored to specific research objectives. Process mass balance calculations are prioritized for kinetic investigations; solid-phase quantification serves as the core strategy for evaluating end-product CO_2_ sequestration capacity; mineralogical characterizations such as XRD and FTIR are required to provide complementary evidence for reaction mechanism interpretation.

The seven techniques were selected using three explicit criteria: (i) the method yields a quantitative signal that can be converted directly, or through a defined stoichiometric step, into CO_2_ mass per unit dry slag; (ii) it has recurrent use or sufficient methodological maturity in mineral carbonation and steel slag studies to permit cross-study comparison; and (iii) the combined set spans the principal measurement dimensions gas-phase disappearance, bulk mass change, total or acid-soluble inorganic carbon, combustion-derived carbon, and crystalline carbonate phases. The list is therefore purpose-driven rather than exhaustive. Muffle-furnace thermal decomposition, temperature-programmed desorption coupled with non-dispersive infrared (NDIR) detection, and coulometric detection are treated here as closely related release/detection variants or auxiliary configurations rather than independent carbon pool methods. FTIR, Raman spectroscopy, SEM-EDS, and microscopy are retained mainly as qualitative or semi-quantitative confirmation tools because their signals are strongly matrix- and calibration-dependent. Isotopic tracing and solid-state NMR can provide powerful mechanistic evidence but currently require specialist calibration and are less suitable as baseline methods for routine industrial verification [39,41].

Accordingly, method selection should follow the research objective rather than a universal ranking. Kinetic and reactor-design studies should prioritize gas consumption monitoring but verify the endpoint by an independent solid-carbon method. Absolute stable sequestration claims should prioritize baseline-corrected TIC/elemental analysis, acid digestion, TGA, or validated combustion-infrared analysis, preferably using at least two independent principles. Engineering-scale screening of blocks or aggregates may use mass gain for trends, but absolute values require solid-phase confirmation. XRD and FTIR are most appropriately used to constrain mineralogical pathways and to explain discrepancies rather than to certify total CO_2_ sequestration capacity independently.

### 3.1. Thermogravimetric Analysis

Among all quantification approaches for the CO_2_ sequestration capacity of steel slag, thermogravimetric analysis (TGA) represents the most widely adopted technique. This method calculates carbonate content in specimens based on mass loss induced by CO_2_ release during thermal decomposition of carbonates within characteristic temperature windows [38,57,58]. For steel slag samples, low-temperature mass loss generally originates from evaporation of free water and dehydration of C-S-H gels; mass reduction ranging from 400 to 550 °C corresponds to dihydroxylation and decomposition of Ca(OH)_2_, whereas mass loss recorded at 550–850 °C is attributed to thermal breakdown of carbonate phases. When the dry mass measured at 150 °C is defined as the baseline, the mass loss detected between 550 and 850 °C can be converted into the CO_2_ content, which is further used to calculate the CO_2_ sequestration capacity of steel slag. The corresponding calculation formulas are expressed as Equations (1) and (2):

(1) $${CO}_{2}\left[wt.\%\right]=\frac{\Delta m_{550–850{}^{\circ}C}\;\left[g\right]}{m_{105{}^{\circ}C}\;\left[g\right]}\times 100\%$$

(2) $$CaCO_{3}\left[wt.\%\right]=\frac{\Delta m_{550–850{}^{\circ}C}\;\left[g\right]}{m_{105{}^{\circ}C}\;\left[g\right]}\times 100\%$$

where Δ*m*_550°C_ denotes the mass of steel slag at 550 °C, g; Δ*m*_850°C_ denotes the mass of steel slag at 850 °C, g; and Δ*m*_105°C_ denotes the mass of steel slag at 105 °C, g; 44.01 refers to the molar mass of CO_2_; and 100.09 represents the molar mass of calcium carbonate.

Compared with other quantification techniques, TGA exhibits outstanding advantages of superior measurement precision [59]. Moreover, this method benefits from widespread instrument availability, minimal sample consumption, and simultaneous acquisition of data regarding dehydration, dihydroxylation, and carbonate decomposition; differential thermogravimetry (DTG) peaks further enable the identification of reaction products formed at distinct thermal stages [60]. In practical testing, temperature boundaries should not be rigidly fixed. The thermal decomposition of carbonate phases, dehydration of C-S-H gels, and breakdown of sulfate phases in carbonated steel slag may occur within overlapping temperature intervals. The major controversial issue concerning TGA quantification lies in the determination of carbonate decomposition temperature windows [61]. In steel slag matrices, thermal events associated with C-S-H, Mg/Fe-bearing phases, sulfate phases, and poorly crystalline carbonates tend to overlap with the decomposition range of CaCO_3_, introducing systematic deviations in final results contingent on the selected temperature intervals [39,62,63,64]. Additionally, the apparent decomposition temperature of carbonates is susceptible to testing parameters, including purge atmosphere, heating rate, sample loading mass, and crucible type. Quantification results lack inter-laboratory comparability if only a fixed temperature range is adopted without full documentation of experimental conditions.

Furthermore, TGA tests require milligram-scale specimens, whereas the intrinsic heterogeneity of steel slag renders such tiny subsamples unrepresentative of bulk slag, failing to reflect the overall CO_2_ sequestration capacity of the entire slag batch [40]. Multiple parallel measurements are therefore mandatory to mitigate localized sampling errors. For monolithic specimens obtained from dry carbonation, parallel tests on subsamples extracted from different positions of the bulk material are required to draw reliable conclusions. Blank tests are also necessary to quantify native carbonate content in raw steel slag, whose value should be subtracted from the total measured carbonate mass to eliminate background interference [65].

### 3.2. Mass Gain Method

The mass gain method quantifies CO_2_ sequestration capacity by calculating the dry mass difference in specimens before and after carbonation [32]. This technique is widely applied to molded monoliths, aggregates, pressed bricks, and semi-dry carbonation curing systems [66], and the corresponding calculation formula is shown in Equation (3) [67]:

(3) $$Q_{CO_{2}}=\frac{W_{1}-W_{0}}{W_{0}}\times 1000\;\left(g\;CO_{2}/kg\;slag\right)$$

where *W*_0_ refers to the mass of raw steel slag dried to constant weight before carbonation, g; *W*_1_ denotes the mass of carbonated steel slag dried to constant weight after reaction, g; *Q*_CO2_ represents the CO_2_ sequestration capacity of steel slag, with the unit of g CO_2_ per kg steel slag.

The mass gain method features simplicity, straightforward operation, and low testing cost, which are suitable for investigating the macroscopic CO_2_ sequestration trends affected by variables including particle size, moisture content, CO_2_ concentration, and carbonation duration [68]. Benefiting from its facile calculation workflow, direct weighing can characterize the overall mass variation of carbonated blocks, pressed slag bricks, and carbonated aggregates, eliminating the representativeness bias induced by microscale sampling [69].

Beyond these gravimetric interferences, carbonation kinetics directly control the reliability of mass-gain-derived CO_2_ uptake. Relative humidity and moisture content determine the water-film conditions required for CO_2_ dissolution and ionic transport: insufficient moisture limits CO_2_ dissolution and reaction, whereas excessive moisture can hinder gaseous CO_2_ diffusion through the pore network. CO_2_ concentration, temperature, particle size, porosity, and carbonation duration further alter the reaction rate and extent by changing gas transfer, Ca/Mg leaching, accessible reactive surface area, and product-layer growth. Therefore, the mass gain method should be interpreted as quantifying the CO_2_ sequestration achieved under an explicitly specified set of carbonation and drying conditions, rather than the intrinsic total carbonation capacity of the material. Only when reaction completion is independently verified, and contributions from moisture, hydration, residual salts, dissolution, and particle loss are eliminated, can a mass-gain result be used to approximate the total attainable CO_2_ sequestration capacity.

The most critical limitation of this method lies in the oversimplified equivalence between total mass increment and actual CO_2_ sequestration. For carbonation under wet, semi-dry, or molded specimen conditions, mass fluctuation can be triggered by moisture adsorption, hydration reactions, residual salts, fine particle loss, and migration of dissolved constituents, and such mass variations may reach the same magnitude as CO_2_-induced mass gain [70]. Accordingly, the mass gain method is only applicable to internal trend screening within identical reaction systems and cannot serve as primary evidence for absolute CO_2_ sequestration quantification. Without standardized constant-weight drying protocols, blank hydration control groups, and supplementary solid-phase carbon analysis for calibration, the sole adoption of the mass increment ratio to calculate CO_2_ sequestration capacity is methodologically inadequate.

### 3.3. Gas Consumption Method

The gas consumption method calculates the net consumption of gaseous CO_2_ throughout carbonation reactions by monitoring real-time variations in CO_2_ concentration, gas flow rate, pressure, temperature, or reactor volume [71]. Its irreplaceable advantage lies in the acquisition of time-resolved kinetic curves. This technique enables in situ, continuous, and dynamic monitoring of carbonation progression and allows precise determination of critical kinetic parameters such as CO_2_ sequestration rate and reaction equilibrium point, which provide fundamental data support for the optimization of carbonation operating conditions. It can distinguish sequential reaction stages, including rapid surface reaction, diffusion-limited reaction, and reaction passivation, and further support reactor design, process scale-up, and dynamic mechanistic analysis of CO_2_ sequestration.

Notably, this method quantifies the disappearance of CO_2_ from the gas phase rather than the permanent incorporation of CO_2_ into solid matrices as thermodynamically stable carbonates. The major drawback is that temporary storage of DIC in the liquid phase, entrapped CO_2_ within pores, sensor drift, and system leakage errors are readily misidentified as mineralized CO_2_ sequestration. If only cumulative CO_2_ consumption is reported without auxiliary calibrations including blank reactor leakage tests, inert reference sample correction, liquid-phase DIC measurement, and cross-verification via solid-phase total inorganic carbon (TIC), TGA, or acid digestion, the obtained value merely represents apparent CO_2_ uptake within the reactor rather than authentic CO_2_ sequestration capacity, and thus cannot be directly adopted for carbon emission reduction accounting.

### 3.4. Acid Digestion Method

The acid digestion method is a quantification technique for CO_2_ sequestration capacity built upon classical chemical analysis principles. Its core mechanism lies in the liberation of CO_2_ via chemical decomposition reactions between acid solutions and carbonate phases [72]. The mass of released CO_2_ is quantified through gas volume measurement, pressure variation recording, absorbent solution titration, or infrared detection to calculate the total calcium carbonate content in specimens [39]. Unlike TGA, this method requires no predefinition of thermal decomposition temperature windows; compared with XRD, it is capable of capturing substantial amorphous or poorly crystalline carbonates. The acid digestion method achieves more consistent quantification of carbonate contents in multiphase specimens and has gained extensive attention in recent quantitative studies on CO_2_ sequestration within cementitious materials [73].

The irreplaceable value of acid digestion in the multi-layer evidence chain is that it provides chemical verification of total acid-soluble inorganic carbonates and mitigates sampling errors arising from local sample heterogeneity. For steel slag containing hydration products, poorly crystalline carbonates or overlapping thermal decomposition peaks, acid digestion serves as a powerful cross-calibration tool for TGA and compensates for the undetected amorphous carbonate fractions in XRD measurements [42]. Furthermore, the strong acid adopted in this protocol reacts selectively with inorganic carbonates in the sample, and organic carbon fractions will theoretically not be incorporated into the quantified carbon value.

Although the acid digestion method approximates the measurement of total carbonate content, its reliability is not unconditional. The evolved gas is not exclusively CO_2_: sulfur-rich steel slag may generate H_2_S upon exposure to strong acid, and metallic or reductive mineral fractions can trigger side reactions, which necessitate corresponding corrections in CO_2_ quantification [38]. Incomplete acid digestion may occur for coarse particles, carbonates encapsulated by silica gel, or dense carbonate product shells. Additionally, partial dissolution of CO_2_ in absorption liquids or water-sealed setups leads to underestimated carbon values. All the above factors govern the credibility of final test results. Accordingly, the acid digestion test must be implemented with calcium carbonate standard reference materials, raw slag blank control groups, parallel duplicate specimens, and gas selectivity calibration. If only water displacement, gas collection, or pressure monitoring is adopted without compositional validation of evolved gas, errors induced by chemical side reactions will be introduced into the calculated CO_2_ sequestration capacity.

### 3.5. Elemental Analysis

Elemental analysis ranks among the most precise and widely adopted techniques for quantifying the CO_2_ sequestration capacity of steel slag [74,75]. Its fundamental principle relies on measuring variations in total carbon (TC), total organic carbon (TOC), or TIC of specimens before and after carbonation, and converting the incremental inorganic carbon content into sequestered CO_2_ mass [76]. The corresponding calculation formulas are presented as Equations (4) and (5):

(4) $$m_{bc}\times {CO}_{2,initial}+m_{bc}\times {CO}_{2,uptake}=m_{ac}\;\times \;{CO}_{2,finial}$$

(5) $$CO_{2}=\frac{CO_{2,uptake}}{m_{ss}}$$

where *m*_bc_ denotes the dry mass of the sample before carbonation, g; *m*_ac_ denotes the dry mass of the sample after carbonation, g; *m*_CO2_ initial denotes the mass percentage of CO_2_ in dry samples before carbonation, %; *m*_CO2_ final denotes the mass percentage of CO_2_ in dry samples after carbonation, %; *m*_ss_ denotes the mass percentage of steel slag in the raw sample, %.

The irreplaceable merit of elemental analysis lies in its capacity to establish a full carbon mass balance ledger for the system [77]. For specimens containing amorphous carbonates, low-concentration carbonate phases, or carbon species undetectable by XRD, the increment of TIC better reflects the total bound carbon compared with crystalline-phase quantification. When combined with major elemental analysis techniques such as XRF or ICP, this method can identify leaching or migration of Ca, Mg, and other metallic cations, eliminating the misjudgment that an elevated carbon mass fraction per unit solid mass equals net CO_2_ sequestration gain.

Elemental analysis possesses the highest priority among all quantification approaches, yet it strictly targets incremental inorganic carbon rather than undifferentiated total carbon. If raw native carbonates, organic admixtures, grinding aids, carbon powder, or residual solvents exist in samples, reporting only TC increments will introduce systematic overestimation of mineralized CO_2_. Cheng et al. [78] quantified carbonation degrees of cement pastes via an elemental analyzer based on total carbon measurement, and corrected background biases originating from raw steel slag through blank control tests. Standardized operation requires raw slag from the same batch to be set as the baseline, separate quantification of TC, TOC, and TIC, and integration of mass variations before and after carbonation into a unified dry-basis mass balance framework. Otherwise, the superior precision of elemental analysis merely amplifies errors derived from sample pretreatment and carbon speciation discrimination and cannot guarantee authentic CO_2_ sequestration data independently. Coupling elemental analysis with XRF or ICP enables verification of major elemental mass conservation, substantially improving the reliability of CO_2_ sequestration calculations.

### 3.6. Combustion-Infrared Absorption Method

Infrared spectroscopy can acquire molecular information of various species within specimens, which has been adopted for quantitative analysis of carbonation depth [41]. For the combustion-infrared absorption method, carbon contained in samples is fully converted into CO_2_ under high-temperature oxygen flow, and the generated CO_2_ is quantified by an infrared detector according to its characteristic absorption intensity [42]. This technique features rapid testing speed, high detection sensitivity, and superior automation, making it suitable for high-throughput sample screening, industrial quality control, and standardized laboratory testing. Unlike TGA, it eliminates artificial partitioning of complicated thermal decomposition intervals; compared with acid digestion, high-temperature oxidation or decomposition can completely release CO_2_ from encapsulated and poorly soluble carbonates. Such merits render the combustion-infrared method highly practical for batch sample screening, industrial quality control, and standardized laboratory operations [39].

The combustion-infrared absorption method exhibits excellent instrumental repeatability and serves optimally for the rapid quantification of total carbon. Nevertheless, its core limitation lies in the mismatch between high detection sensitivity and low mineralization selectivity. Any organic carbon, free elemental carbon, carbon-containing admixtures, or exogenous carbon contaminants in the specimen will be oxidized into CO_2_ during combustion and incorporated into the measured carbon value [79]. Accordingly, this method must be coupled with acidification pretreatment, TOC/TIC separation, or raw slag background subtraction. Direct conversion of total carbon measured via combustion-infrared analysis into CO_2_ sequestration capacity essentially attributes all carbon sources to CO_2_ mineralization, which is unacceptable for carbon emission reduction accounting.

### 3.7. XRD Method

The XRD method is an approach for CO_2_ sequestration capacity determination based on crystalline diffraction properties [80]. Its core principle adopts Rietveld refinement via software packages, including TOPAS-Academic 7 and HighScore Plus 4.0, to fit X-ray diffraction patterns and quantify crystalline mineral phases within specimens [81,82,83]. The CO_2_ content is subsequently converted from the chemical formulas of carbonate phases to calculate the CO_2_ sequestration capacity of steel slag [82]. By quantitatively tracking mineralogical phase evolution, XRD can elucidate the transformation relationships among f-CaO, Ca(OH)_2_, C_2_S, C_3_S, calcite, aragonite, vaterite, and other phases throughout carbonation [84,85]. Its unique strength does not lie in superior measurement precision, but in its capacity to interpret reaction pathways: it identifies which reactive mineral phases participate in carbonation, which crystalline carbonate polymorphs form, and whether unreacted reactive phases remain, thereby supporting the prediction of relevant material performances [86]. In addition, XRD enables assessment of carbonation completeness. The diffraction peak intensities of residual reactive components such as unreacted CaO and Ca(OH)_2_ indirectly reflect the extent of carbonation reactions. This renders XRD suitable for comparing carbonation performance under diverse reaction conditions and guiding the optimization of carbonation process parameters. For instance, simultaneous growth of calcite alongside consumption of C_2_S verifies the participation of C_2_S in carbonation reactions; attenuation of diffraction peaks corresponding to free CaO indicates a remarkable reduction in volume expansion instability risks [74].

Nevertheless, quantitative XRD results cannot be equated to the total CO_2_ sequestration capacity of steel slag. The primary limitation of this technique is its insensitivity to amorphous or poorly crystalline carbonates [87]. Even with internal standard calibration and Rietveld refinement, the derived data only represent crystalline carbonate fractions rather than the total inorganic carbon pool. Glassy phases, iron-bearing phases, and RO phases inherent to steel slag cause severe diffraction peak overlap. Furthermore, preferred orientation of particles, grinding fineness, and instrumental setup parameters all exert adverse impacts on quantification accuracy. Accordingly, studies reporting absolute CO_2_ sequestration values solely from XRD-Rietveld refinement lack sufficient supporting evidence. Supplementary solid-phase total carbon quantification techniques such as acid digestion, TIC measurement, or TGA are mandatory for cross-calibration. In particular, when the measured TIC value substantially exceeds the carbon content converted from crystalline carbonate quantification, the potential contribution of amorphous carbonates should be explicitly stated, and lower weighting should be assigned to XRD-derived data. In summary, XRD serves optimally as a phase composition constraint technique rather than an independent, standalone method for determining total CO_2_ sequestration capacity.

**Table 1:** Comparison of common quantitative characterization methods for CO_2_ sequestration via mineral carbonation (slag).

Method	Direct Measurement Signal	Key Advantages	Key Limitations	Overestimation Risks	Underestimation Risks	Ref.
TGA	Mass loss during heating/DTG peaks	Identifies characteristic weight loss intervals of carbonates and bound water; key evidence for judging carbonate formation and thermal stability	Overlapping temperature intervals; small sample size	Dehydration/dihydroxylation misattributed to CO_2_	Amorphous/low-temperature decomposable carbonates are underestimated	[74,88,89,90]
Mass gain method	Net mass change before and after carbonation	Directly reflects net mass gain of samples before and after carbonation; serves as a preliminary macroscopic indicator of CO_2_ sequestration changes	Significant interference from moisture and dissolved salts	Hygroscopic moisture, hydration, salt residue	Dissolution and particle loss offset mass gain	[49,83,91]
Gas consumption method	Gas-phase CO_2_ concentration/pressure/flow rate changes	Enables in-line characterization of CO_2_ consumption; provides important kinetic data for analyzing absorption rate, reaction stages, and endpoint	Dissolved CO_2_ and temperature–pressure effects are difficult to decouple	Transient liquid-phase storage; T/P calibration errors	Outlet lag; sensor drift	[48]
Acid digestion method	CO_2_ volume/pressure/titration quantification upon acid release	Quantifies acid-soluble carbonates via CO_2_ release upon acid dissolution; direct chemical evidence for verifying solid-phase carbonate formation	Side reactions and incomplete acid digestion	H_2_S and other by-product gases	Encapsulated carbonates; coarse particles undigested	[92,93,94]
Elemental analysis	TC/TOC/TIC and major elemental variations	Distinguishes total carbon, inorganic carbon, and organic carbon; the core basis for establishing the carbon budget and identifying carbon sources	Speciation depends on sample pretreatment	Organic carbon/free high-carbon residue	Incomplete acid removal or sample segregation	[28,95]
Combustion-infrared method	CO_2_ infrared absorption after combustion	Suitable for rapid quantification of total or inorganic carbon; enables multi-sample carbon content comparison and carbon budget verification	Total carbon and inorganic carbon require differentiation	Organic carbon additives; carbonaceous residue	Incomplete combustion; improper calibration	[42]
XRD	Crystalline phase diffraction peaks and Rietveld quantification	Determines carbonate crystal polymorphs and residual minerals; structural evidence for verifying phase composition of CO_2_ sequestration products	Amorphous phases undetected; peak overlap	Rietveld model errors	Amorphous carbonates and low-content carbonates are underestimated	[55,96,97,98]

### 3.8. Operational Carbon-Pool Taxonomy and Method Correspondence

To prevent the term CO_2_ sequestration capacity from referring to incompatible physical quantities, this review adopts an operational carbon-pool taxonomy. Table 2 summarizes these operational carbon pools, measurable process observables, and their mapping relationships with the seven core quantification techniques for steel-slag CO_2_ sequestration. Let *C*_0_ denote pre-existing inorganic carbon in the raw slag; *C_s_*_,_*_new_* the newly retained stable solid inorganic carbon after intentional carbonation; *C_l_* the dissolved inorganic carbon (DIC) remaining in process liquid, condensate, or washings; *C_p_* physically retained or pore-entrapped CO_2_; and *C_x_* the crystalline-carbonate carbon quantified by XRD, which is a subset of *C_s_*_,_*_new_*. The process observables *G* (net gas-phase CO_2_ disappearance after blank correction) and *M* (dry solid mass change) are not themselves carbon pools. In an ideal closed system, *G* = *C_s_*_,_*_new_* + *C_l_* + *C_p_*. In a real system, a residual term *ε_sys_* must be retained for leakage, sensor drift, and unmeasured transfers. Crystalline-phase quantification obeys *C_x_* ≤ *C_s_*_,_*_new_*; the difference *C_s_*_,_*_new_* − *C_x_* represents amorphous, poorly crystalline, or otherwise XRD-invisible stable inorganic carbon. Likewise, only after correction for hydration, residual moisture and salts, dissolution, leaching, and particle loss should *M* converge toward *C_s_*_,_*_new_*. These relationships explain why nominally identical “CO_2_ sequestration capacity” values from different methods are comparable only when their non-target pools and correction terms are negligible or independently quantified.

## 4. Comparative Analysis and Origin Interpretation of Discrepancies Among Quantification Approaches

The fundamental discrepancy across diverse quantification methodologies originates from the distinct carbon pools targeted by each measurement technique. Four characteristic indicators, namely gaseous CO_2_ consumption, solid mass increment, incremental inorganic carbon in carbonated products, and crystalline carbonate content, are only equivalent under idealized conditions, while systematic deviations persist in practical steel slag carbonation systems.

Sample pretreatment and handling should be treated as controlled experimental variables rather than neutral preparation steps. Five factors require explicit evaluation. (i) Drying temperature and atmosphere determine the residual water baseline and can also alter hydration products or shift overlapping H_2_O/CO_2_ release; samples should therefore be dried to a documented constant-mass endpoint using a procedure validated for the target matrix, with temperature, pressure/atmosphere, duration, and cooling conditions reported [39]. (ii) Grinding fineness improves homogenization and acid accessibility but can change XRD preferred orientation, expose fresh surfaces to atmospheric CO_2_, and alter recovery from encapsulated carbonates; particle-size distribution and grinding duration should be reported, and fineness recovery checks are recommended when acid digestion and TGA results diverge [38,40,42]. (iii) Storage duration and humidity can increase native carbonation before the experiment, requiring a contemporaneous raw slag baseline. (iv) Washing can remove soluble salts and DIC but may also remove fine carbonate precipitates; if washing is part of the process, the wash liquor should be analyzed for inorganic carbon and included in the system boundary. (v) Exposure of dried or finely ground samples to ambient CO_2_ between preparation and measurement should be minimized by sealed storage and timed transfers because this exposure directly modifies the baseline.

A minimum reproducibility protocol is therefore recommended: homogenize a sufficiently large parent sample; split matched aliquots for all analytical methods; apply the same dry basis definition; record drying, grinding, washing, storage, and ambient exposure histories; include raw slag and process blanks; and perform paired sensitivity checks whenever the matrix is expected to be pretreatment sensitive. Pretreatment-induced differences should be propagated as uncertainty components rather than absorbed into an undifferentiated method error. This approach is consistent with recent evidence that moisture control, grinding, acid accessibility, and overlapping thermal events can produce systematic rather than random deviations [39,42].

Taking the work reported in Reference [42] as an illustration, four quantification techniques were adopted to determine the CO_2_ sequestration capacity of identical slag specimens, and the 7-day CO_2_ sequestration capacity results are presented in Figure 4. Importantly, the four determinations shown in Figure 4 were obtained from the same steel slag specimens subjected to identical carbonation conditions, so the observed numerical spread primarily reflects differences among the CO_2_ quantification procedures rather than differences in carbonation history. The CO_2_ sequestration capacity derived from stepwise thermogravimetry (Stepwise-TG) reached 9 g/100 g slag, whereas the value obtained via tangential thermogravimetry (Tangential-TG) was only 6.5 g/100 g slag. The combustion-infrared method (AD-TIC) yielded a value of 10.2 g/100 g slag, and the acid digestion method (HT-TIC) produced 9.7 g/100 g slag. The experimental data revealed that deviations between different measurement methods could exceed 30%. Relative to the highest value (10.2 g CO_2_/100 g), the tangential-TG result is 36.3% lower, the stepwise-TG result is 11.8% lower, and the HT-TIC result is 4.9% lower; the absolute highest-to-lowest spread is 3.7 g CO_2_/100 g. Thermogravimetric analysis evidently underestimates the actual CO_2_ sequestration capacity, and the remaining quantification approaches exhibit relatively consistent results with each other. The primary contributor to such divergence lies in substantial uncertainty induced by subjective selection of thermal decomposition temperature windows for TGA measurements. In addition, acid digestion generally delivers lower CO_2_ sequestration values than the combustion-infrared method. This phenomenon can be attributed to partial dissolution of evolved CO_2_ in liquid media and encapsulation of fine calcium carbonate particles within gel phases, which hinder complete acid dissolution and impair quantitative accuracy [79,99]. Such numerical divergences do not inherently indicate that a certain method generates erroneous data; instead, they reflect divergent responses of each technique to thermal decomposition cut-off temperatures, acid digestion completeness, sample background signals, and carbonate speciation.

Baciocchi et al. [100] further compared steel slag carbonation via thin-film and aqueous slurry routes. Under slurry-phase conditions at 100 °C and a CO_2_ partial pressure of 10 bar, the maximum CO_2_ sequestration capacities of EAF, BOF1, and BOF2 steel slags were 280, 325, and 403 g CO_2_ per kg slag, respectively. The CO_2_ sequestration capacity of BOF2 slag was 123 g CO_2_/kg higher than that of EAF slag, corresponding to an increment of approximately 43.9%. Nevertheless, it should be emphasized that such numerical gaps stem not merely from inherent mineralogical variations among slag types but also strongly rely on the implicit quantification boundaries embedded within the adopted measurement strategy. This study calculated CO_2_ sequestration capacity based on recorded gaseous CO_2_ consumption in slurry systems rather than solid mass gain and carbonation processes. Accordingly, both quantification approaches and defined calculation boundaries must be fully documented when comparing the performance of carbonation technologies based on the published literature. Failure to provide such information will trigger misattribution of test results, blur the independent influences of slag category, reaction parameters, and quantification schemes, and ultimately undermine the scientific validity of process evaluation.

The two representative cases above distinguish two categories of measurement discrepancies. The first category refers to method-induced deviations generated from identical raw materials measured via different quantification protocols, which necessitates cross-verification with multiple complementary testing techniques. The second category denotes system-dependent deviations arising from the coupling of variable slag feedstocks/carbonation routes and quantification methodologies, which requires a unified sample background, standardized reaction parameters, and consistent reporting units for comparative analysis. Confusion between these two types of discrepancies will eliminate the comparability of CO_2_ sequestration data reported across different publications.

For future inter-method validation, the seven investigated quantification methods should, wherever analytically compatible, be applied to identical or homogenized aliquots from the same carbonated steel slag batch produced under one controlled carbonation regime. All results should be converted to a common dry basis unit (g CO_2_/kg raw slag) and use matched sample pretreatment and raw slag background subtraction. This same sample design would allow pairwise and overall differences in reported CO_2_ sequestration capacity to be attributed systematically to the analytical method rather than to feedstock heterogeneity or carbonation-condition variability.

Table 3 summarizes the origins, manifestations, and corrective strategies of discrepancies among quantification approaches. As illustrated in Table 3, overestimation risks are mostly associated with extraneous mass or non-mineralized CO_2_, including moisture adsorption, hydration products, residual salts, organic carbon, liquid-phase DIC, and gas pipeline calibration errors. Such interferences frequently occur in process-based, mass-gain, and total-carbon quantification methods. By contrast, underestimation risks mainly arise from narrow identification boundaries or incomplete release of carbonate-bound CO_2_. Typical examples include undetected amorphous carbonates via XRD, inaccessible carbonates encapsulated by silica gel or product layers during acid digestion, and unaccounted complex carbonate phases caused by overly narrow temperature windows in TGA testing. The mirror-like distribution of overestimation and underestimation risks establishes the theoretical foundation for collaborative quantification via multiple complementary methods. If results from gas-phase measurements greatly exceed solid-phase data, liquid-phase DIC storage and system leakage should be prioritized for inspection. If XRD-derived values are markedly lower than TIC or TGA results, the presence of amorphous or poorly crystalline carbonates is highly probable. Where mass gain outputs exceed acid digestion or TIC measurements, residual moisture and soluble salts constitute the primary interference sources requiring elimination.

Accordingly, discrepancies between different quantification techniques should not be merely simplified as broad error bars, but interpreted as indicators of an unresolved carbon pool mass balance. High-quality research is expected to transparently report all divergent datasets rather than selectively adopt a single favorable value. Only after such discrepancies are clearly attributed, systematically corrected, or incorporated into expanded uncertainty evaluation can CO_2_ sequestration data achieve cross-study comparability and be applicable for industrial carbon verification.

## 5. Framework for Collaborative Quantification via Multiple Analytical Methods

Owing to the incommensurability and insufficient accuracy of CO_2_ sequestration capacity relying solely on a single testing technique [79], this study integrates the respective merits, inherent limitations, and discrepancy origins of all aforementioned approaches and proposes a paradigm shift for steel slag CO_2_ sequestration quantification from single-method readout to multi-method evidence chain validation. The proposed evidence chain does not merely mean an increase in testing replicates; instead, different analytical techniques are deployed to characterize distinct carbon pools at separate dimensions, forming a closed-loop verification system following the sequential workflow of baseline characterization, process mass balance, solid-product carbon confirmation, and statistical fusion. Step-by-step construction of traceable evidence chains ensures that quantified CO_2_ sequestration data feature well-defined calculation boundaries, cross-method mutual verification, and quantifiable measurement uncertainty. The entire framework is hierarchically divided into four modules: raw material baseline layer, reaction process mass balance layer, solid product carbon quantification layer, and statistical fusion layer. Table 4 presents the standardized workflow of multi-method collaborative quantification for the CO_2_ sequestration capacity of steel slag.

To make the four-layer framework operational, each layer passes a defined data object and a decision flag to the next layer, and Figure 5 presents the decision framework of the four-layer evidence chain. Layer 1 outputs *C*_0_ together with dry-basis mass, composition, particle-size, storage, and pretreatment metadata. Layer 2 receives that baseline and outputs corrected gas consumption *G*, process conditions, liquid/solid carbon terms where available, and the mass-closure residual. Layer 3 outputs at least two independent estimates of *C_s_*_,_*_new_* together with mineralogical constraints from XRD/FTIR. Layer 4 receives only datasets that pass the preceding quality gates and reports either a fused value with expanded uncertainty or an explicitly bounded method-dependent range. Gate A (after Layer 1) requires a documented baseline and common dry basis; Gate B (after Layer 2) requires acceptable closure or a physically assigned discrepancy; Gate C (after Layer 3) requires cross-method agreement within the acceptance band or activation of a third method. Failure at any gate returns the workflow to the source-specific diagnostic step rather than permitting unconditional averaging. In this manner, information flows forward only with retained provenance, while every correction remains traceable backward to its originating layer.

The first layer: raw material baseline layer. The core objective of this layer is to quantify the initial carbon background of raw steel slag, which serves as the zero baseline for subsequent CO_2_ sequestration quantification. Natural carbonation may occur during stockpiling, crushing, and pretreatment of steel slag. Failure to subtract such background carbon will directly lead to overestimation of net CO_2_ sequestration capacity generated in carbonation experiments. Accordingly, before any CO_2_ sequestration measurement, the TIC and LOI of raw steel slag should be fully documented, together with detailed records of sample drying protocols, particle size ranges, sampling strategies, and replicate testing numbers. The TIC value of uncarbonated slag defines the initial inorganic carbon background, while LOI assists in evaluating combined interferences from free moisture, hydroxides, carbonates, and other volatile constituents within specimens. On this basis, the net CO_2_ increment of solid carbonated products shall be calculated via differential computation under a unified dry basis and consistent testing specification, as expressed in Equation (6):

(6) $$\Delta m_{CO_{2}}=m_{CO_{2},product}-m_{CO_{2},initial}$$

where Δ*m*_CO2_ denotes the net mass of newly formed CO_2_ in solid phases after carbonation, g; *m*_CO2,product_ represents the total CO_2_ mass contained in carbonated specimens, g; *m*_CO2,initial_ is the background CO_2_ mass inherently existing in untreated raw steel slag. If the measured TIC is expressed in mass of elemental carbon, a conversion factor of 44.01/12.01 shall be adopted to convert carbon mass into equivalent CO_2_ mass. The primary function of this layer is not to output the final CO_2_ sequestration value but to establish a unified baseline for raw feedstock, preventing native carbonates in original slag from being falsely counted as newly mineralized CO_2_ sequestration products.

For steel slags with an aging or storage history, background correction must be batch-, size fraction, and time-specific. The baseline specimen should be taken after the same crushing, grinding, drying, and washing steps used for the test material and as close as practicable to the start of intentional carbonation; it should not be inferred from nominal fresh-slag composition or transferred from a different stockpile date. For aged or outdoor-stored slags, the report should include storage duration, cover/ventilation condition, moisture history, and particle size distribution, and the as-received native TIC should be determined on replicate homogenized aliquots when heterogeneity is appreciable. If the storage history is unknown, the result should be labeled “net CO_2_ sequestration capacity above the measured as-received baseline” rather than total sequestration since slag production. Washing, thermal treatment, or other pretreatments that remove carbon-bearing species change *C*_0_ and therefore require a new baseline measured after that step. Baseline transfer between different slag batches is discouraged. This procedure distinguishes newly sequestered CO_2_ from inherited carbonate carbon without assuming a known historical carbonation trajectory.

The second layer: process mass balance layer. The core mission of this layer is to record the input, output, consumption, and residual distribution of CO_2_ throughout the carbonation reaction system. For autoclave closed systems, total CO_2_ consumption can be calculated based on variations in pressure, temperature, reactor volume, and gas composition. For continuous flow reactors, cumulative CO_2_ sequestration capacity is obtained by integrating inlet and outlet CO_2_ concentrations, gas flow rates, and reaction duration. For carbonation chambers or molded component curing systems, real-time records of CO_2_ concentration, relative humidity, temperature, and specimen mass variation are also required. It must be emphasized that the CO_2_ consumption quantified in the gas phase only reflects the reduction in gaseous CO_2_, which cannot be directly equated to permanently mineralized CO_2_ sequestration capacity. A fraction of CO_2_ may remain as dissolved inorganic carbon (DIC) in liquid phases, be trapped within pore structures, escape via system leakage, or generate biased readings due to sensor drift.

To enhance the reliability of process mass balance calculations, a mass closure check is recommended at this layer. The closure deviation can be quantified via Equations (7) and (8):

(7) $$\delta \left(\%\right)=\frac{m_{gas\;consumption}-m_{mass\;gain}}{m_{gas\;consumption}}\times 100\%$$

(8) $$\left|\delta \right|=\left|\frac{m_{gas\;consumption}-m_{mass\;gain}}{m_{gas\;consumption}}\times 100\%\right|$$

where *m*_gas consumption_ refers to the cumulative mass of consumed CO_2_ measured from the gas phase, g; *m*_mass gain_ stands for the net CO_2_ mass increment of solid products, g. The sign of this deviation index indicates the direction of mass imbalance. If the gas-phase CO_2_ consumption is markedly higher than the solid-phase CO_2_ increment, unaccounted liquid-phase DIC, gas pipeline leakage, pore-entrapped CO_2_, or sensor drift are highly probable causes. Conversely, when the solid-phase carbon gain exceeds the gas-phase consumption, priority shall be given to verifying background subtraction of raw slag, drying protocols, sampling representativeness, and conversion criteria adopted for elemental analysis.

For reproducible decision-making, mass closure deviations are interpreted in three bands. |δ| ≤ 10% is proposed as the target acceptance range for laboratory-scale quantification; 10% < |δ| ≤ 15% is a conditional warning range in which a provisional value may be reported only with identified correction terms and expanded uncertainty; and |δ| > 15% is considered failed closure and precludes a single reconciled sequestration value. These percentages are operational decision limits proposed by this review rather than an established international metrological standard, and they should be refined through future interlaboratory validation. Conflicting results should be resolved in a fixed hierarchy: (1) verify *C*_0_ and dry basis normalization; (2) audit gas leakage, sensor calibration, DIC, condensate, and other phase transfers; (3) check method-specific recovery, blank correction, and sample homogenization; (4) use XRD/FTIR to diagnose crystalline amorphous mismatches; and (5) if a systematic bias remains unresolved, report the method-specific range rather than calculate a weighted average. The same logic is applied to *D_ij_* in the product layer so that statistical fusion is permitted only after systematic incompatibility has been excluded.

For practical data reporting, the absolute value |δ| is adopted as the criterion for judging mass closure deviation. When the closure deviation exceeds 15%, a single CO_2_ sequestration value shall not be directly reported. Instead, systematic troubleshooting should be carried out, including reactor leakage, liquid-phase DIC, inorganic carbon retained in condensate or washing solutions, sample loss, sampling bias, as well as calibration of gas flow rate and CO_2_ concentration sensors.

The third layer: solid product carbon quantification layer. The core task of this layer is to verify that CO_2_ is permanently incorporated into the carbonated steel slag matrix in the form of carbonate minerals or stable inorganic carbon. This layer serves as the primary basis for final CO_2_ sequestration quantification, rather than merely relying on gas-phase consumption or specimen mass gain. Given the distinct measurable targets and error sources of TGA, acid digestion, elemental analysis, and combustion-infrared absorption methods, this study proposes a minimum verification configuration termed four-point mutual validation. Any research reporting CO_2_ sequestration capacity must provide consistent data from at least two of the four techniques above. The carbon quantification results from solid products are regarded as basically consistent if the relative deviation of net CO_2_ sequestration values from two methods is less than 10%. The relative deviation is calculated following Equation (9):

(9) $$D_{ij}=\frac{\left|Q_{i}-Q_{j}\right|}{\frac{Q_{i}+Q_{j}}{2}}\times 100\%$$

where *Q*_i_ and *Q*_j_ denote the net CO_2_ sequestration capacity acquired by two independent quantification methods, with a unified unit of g CO_2_ per kg raw steel slag. When *D*_ij_ < 10%, favorable consistency between the two solid-phase testing approaches is confirmed. For specimens with complex mineral assemblages or low carbonation extents, if *D*_ij_ ranges from 10% to 15%, a third analytical method shall be introduced to judge the credible carbon value, and the origins of deviation must be explicitly documented in the manuscript. If *D*_ij_ > 15%, weighted averaging is not recommended; instead, the CO_2_ sequestration result shall be reported as a value range (*Q*_min_~*Q*_max_). Comprehensive troubleshooting is required, including rationality of TGA temperature window partitioning, completeness of acid digestion reactions, TIC/TOC separation performance in elemental analysis, sample homogenization efficiency, drying protocols, raw slag background subtraction procedures, and organic carbon elimination efficiency for combustion-infrared tests. If valid data are available from all four core quantification techniques, all four groups of results shall be presented simultaneously, alongside a discussion on systematic overestimation or underestimation trends. Furthermore, supplementary characterization tools, including XRD and solid-state FTIR, serve as mineralogical evidence for reaction mechanism interpretation. They can identify carbonate polymorphs, amorphous phases, residual unreacted reactive minerals, and the microstructure of product layers, yet cannot be adopted as standalone evidence for total CO_2_ sequestration quantification.

The fourth layer: statistical fusion layer. The core objective of this layer is to integrate CO_2_ sequestration data from multiple independent methods into a definitive final value accompanied by quantitative measurement uncertainty, rather than merely outputting an ostensibly precise single number. For each quantification technique, all uncertainty contributions shall be documented as thoroughly as possible, including standard deviation of parallel replicates, instrumental calibration error, sampling error, and model-induced error. Additional uncertainty sources are method-specific: uncertainty arising from subjective temperature window selection for TGA; measurement errors of temperature, pressure, gas flow rate, and CO_2_ concentration for gas-phase approaches; biases from standard reference material calibration, blank correction, and sample homogenization for acid digestion and elemental analysis; interferences induced by organic carbon contained in admixtures throughout carbonation tests for combustion-infrared spectrometry.

If consistent results are obtained across multiple testing methods, weighted averaging can be implemented to calculate the integrated CO_2_ sequestration capacity (Equation (10)). In accordance with the law of error propagation, when all measurement methods are mutually independent, the combined standard uncertainty of the fused result is calculated via Equation (11). The expanded uncertainty is further derived from the combined standard uncertainty (Equation (12)). The final CO_2_ sequestration capacity is expressed in a standardized format with uncertainty (Equation (13)), which is presented in practical writing as: Final CO_2_ sequestration capacity: *X* ± *U* gCO_2_/kg Slag (*k* = 2).

(10) $$X=\frac{\sum_{i=1}^{n}w_{i}Q_{i}}{\sum_{i=1}^{n}w_{i}},w_{i}=\frac{1}{u_{i}^{2}}$$

(11) $$u_{c}=\frac{1}{\sqrt{\sum_{i=1}^{n}\frac{1}{u_{i}^{2}}}}$$

(12) $$U=k\cdot u_{c}$$

(13) $$Q_{finial}=X\pm U$$

where *X* represents the average final CO_2_ sequestration value integrated from multiple methods; *Q*_i_ refers to the net CO_2_ sequestration capacity per unit raw slag measured by the *i*-th testing method; *w*_i_ stands for the statistical weight assigned to the *i*-th quantification method; *u*_i_ denotes the standard uncertainty corresponding to the *i*-th method; *n* means the number of valid testing methods incorporated into data fusion; *u*_c_ represents the combined standard uncertainty after multi-source data integration; *U* indicates the expanded uncertainty; *k* is the standard coverage factor with a fixed value of 2, matching a 95% confidence interval; *Q_fianl_* signifies the ultimate CO_2_ sequestration capacity. If remarkable systematic deviations exist among datasets from different methods, and error sources cannot be identified or corrected, the results should be reported as a value range in the format: CO_2_ sequestration capacity *Q_min_*∼*Q_max_* g CO_2_/kg slag. In addition, the testing methods and quantification boundary constraints corresponding to the upper and lower limits of the range shall be explicitly annotated.

On the basis of the above hierarchical framework, quantification of steel slag CO_2_ sequestration capacity shall abide by the standardized workflow: baseline subtraction first, followed by process mass balance calculation, cross-verification of solid carbon products, and final data reporting via statistical fusion. The raw material baseline layer establishes a definite zero point for net CO_2_ sequestration increment; the process mass balance layer judges whether gaseous CO_2_ consumption maintains mass balance with solid-phase carbon gain; the solid product carbon quantification layer confirms the formation of stable inorganic carbon through mutual cross-verification of TGA, acid digestion, elemental analysis and combustion infrared spectrometry; the statistical fusion layer converts multi-method measurement results into auditable datasets with quantified uncertainty. Only when consistent conclusions are obtained from baseline characterization, process balance, solid product testing, and statistical evaluation can the quantified CO_2_ sequestration capacity of steel slag possess high scientific reliability and a valid engineering carbon verification value.

### 5.1. Robustness Across Steel Slag Types and Matrix-Specific Validation

The proposed framework is intended to be matrix-robust, but it does not imply one universal numerical correction factor for all steel slags. Fixed method-specific correction factors are not recommended because both the magnitude and direction of bias depend on phase assemblage, sulfur and carbon content, hydration state, particle size, and aging. Instead, slag-specific validation is required. In free-CaO/free-MgO-rich BOF or LF slags, hydration and dihydroxylation can distort mass gain and TGA, so blank hydration controls, strict dry basis normalization, and phase-aware thermal windows are essential. In silicate-rich or strongly glassy slags, silica gel and poorly crystalline carbonates increase the risks of incomplete acid digestion and XRD underestimation; grinding recovery tests and TIC/TGA cross-checks should therefore receive higher weight. In ferrite/Fe-rich EAF slags, diffraction peak overlap and acid-induced side reactions require additional Rietveld-quality and gas selectivity checks. Sulfur-rich slags require explicit control of H_2_S interference during acid digestion. Aged or weathered slags require batch- and time-specific *C*_0_ determination because native carbonate can be substantial [27,45,46].

A practical implementation is to classify the feedstock into a matrix-risk profile before selecting methods, using free CaO/MgO, dominant silicate/ferrite phases, sulfur content, LOI/TIC, particle-size distribution, and storage history as descriptors. At least one selected method should be relatively insensitive to the dominant interference: high amorphous content argues against XRD-only quantification; organic or carbonaceous contamination argues against uncorrected combustion total carbon; wet or washed systems require liquid-phase carbon accounting. Consequently, matrix-specific validation procedures are necessary, whereas constant empirical correction coefficients are unlikely to transfer reliably across steelmaking routes.

### 5.2. Worked Multi-Method Case Study Using BOF Steel Slag

A published BOF steel-slag dataset provides a practical worked example of how the proposed evidence chain reconciles real multi-method results. Ferrara et al. [38] analyzed the same carbonated BOF steel slag powder using TG-DTA, acid digestion, and thermal decomposition in a muffle furnace. Reported CO_2_ sequestration capacity values were 15.4%, 14.7%, and 14.3% of the initial slag mass, corresponding to 154, 147, and 143 g CO_2_/kg slag, respectively. The muffle-furnace route is not counted as one of the seven core techniques in this review because it is a thermal-release gravimetric variant, but its use as an auxiliary independent check illustrates why closely overlapping configurations are grouped rather than enumerated as separate core categories.

For Layer 1, the study reports a BOF feedstock containing 40.9% CaO and 5.2% MgO with particle size below 80 μm and uses before/after material characterization; a prospective study under the present framework would additionally archive explicit *C*_0_/TIC and storage metadata. Layer 2 cannot be fully closed from the published dataset because gas-phase, DIC, leakage, and their uncertainty terms are not all reported; therefore, this case is not presented as a complete prospective validation of all four layers. Layer 3, however, passes the proposed criterion: the maximum pairwise relative deviation between 154 and 143 g CO_2_/kg is 7.4%, below the 10% consistency limit. For an illustrative Layer 4 calculation, the equal-weight mean is 148 g CO_2_/kg and the between-method sample standard deviation is 5.6 g CO_2_/kg; using k = 2 gives an expanded dispersion of approximately 11 g CO_2_/kg. An illustrative reconciled report is therefore 148 ± 11 g CO_2_/kg slag (k = 2), while the conservative method-resolved range is 143–154 g CO_2_/kg. Because replicate-level standard uncertainties are not available in the published summary, the ±11 g CO_2_/kg term is explicitly an empirical between-method dispersion rather than a metrologically complete combined uncertainty, and inverse variance weighting would be unjustified. This example demonstrates both the utility and the discipline of the framework: convergent solid-phase evidence can be reconciled, whereas missing process-layer information remains visible instead of being silently imputed.

## 6. Conclusions and Future Perspectives

This paper systematically evaluates the discrepancies among mainstream quantification methods for the CO_2_ sequestration capacity of steel slag. Three core conclusions are drawn as follows. Firstly, the CO_2_ sequestration capacity of steel slag is not an inherent and unique physical parameter. Instead, it is an operational value highly dependent on measurement dimensions and quantification boundaries. Direct equivalence cannot be established among gaseous CO_2_ consumption, solid mass increment, incremental total inorganic carbon, acid-dissolvable carbonate content, and crystalline carbonate content without clear methodological explanations. Secondly, deviations generated by different quantification approaches are not random measurement errors but systematic biases governed by multiple influencing factors, including unclosed carbon pool boundaries, diverse carbonate speciation, initial carbon background of raw steel slag, moisture variation, sample heterogeneity, and instrumental testing modes.

Specifically, the gas consumption method applies to kinetic analysis and reactor carbon mass balance monitoring, while solid-phase mass closure verification is indispensable for reliable results. The mass gain method enables rapid CO_2_ sequestration assessment at the engineering scale, yet interferences originating from moisture adsorption, secondary hydration, and residual soluble salts need to be strictly eliminated. TGA and acid digestion serve as core techniques for solid-phase carbonate quantification, whereas their test accuracy is restricted by subjective temperature window division and incomplete acidolysis reactions, respectively. Elemental analysis and combustion-infrared spectrometry can provide holistic carbon mass balance data, but effective discrimination between organic carbon, inorganic carbon, and raw slag background carbon is a prerequisite for credible results. XRD possesses irreplaceable advantages in identifying carbonate polymorphs and revealing carbonation reaction pathways, but it cannot be adopted independently for the certification of total CO_2_ sequestration capacity.

The expanded framework additionally formalizes the distinction among *C*_0_, *C_s_*_,_*_new_*, *C_l_*, *C_p_*, and crystalline *C_x_*, establishes purpose-based selection criteria for the seven core techniques, and treats pretreatment and storage history as measured sources of uncertainty. Operational decision bands (≤10% acceptable, 10–15% conditional, >15% unresolved) are proposed for closure and cross-method reconciliation, with the explicit caveat that these thresholds require interlaboratory validation. The BOF steel slag case study demonstrates how convergent solid-phase measurements can be reconciled while missing gas/liquid information is flagged rather than inferred. Across distinct slag types, matrix-specific validation is recommended in preference to universal empirical correction factors.

Three priority research directions are proposed for future investigations. First, targeted association standards or industrial standards for steel slag CO_2_ sequestration quantification should be formulated urgently to unify professional terminology specifications, sample pretreatment protocols, matched method combinations, mass closure thresholds, and standardized result reporting formats. Second, an open-access database for carbonated steel slag should be established. Comprehensive information, including raw material mineral compositions, carbonation operating parameters, multi-method CO_2_ sequestration data, measurement uncertainty ranges, and material performance indicators, should be synchronously uploaded, so as to build machine-readable data foundations for cross-study comparison. Third, scale recursive models covering multi-dimensional test scales need to be developed, ranging from milligram-scale tests via TGA and elemental analysis, gram-scale acid digestion tests, kilogram-scale mass gain measurement, to engineering-scale gas mass balance monitoring. Such models can bridge laboratory mechanistic data and field carbon verification outcomes effectively.

Overall, the scientific credibility of steel slag CO_2_ sequestration quantification relies on the closed-loop degree of multi-method evidence chains, rather than a single ideal numerical result. Only when the evidence obtained from reaction process monitoring, solid product detection, and mineralogical characterization is restricted and complements each other, and all measurement uncertainties are reported transparently, can the carbon emission reduction contribution of steel slag carbonation technology be widely recognized by laboratory research systems, practical engineering projects, and authoritative carbon verification institutions.

## Figures and Tables

**Figure 1:**
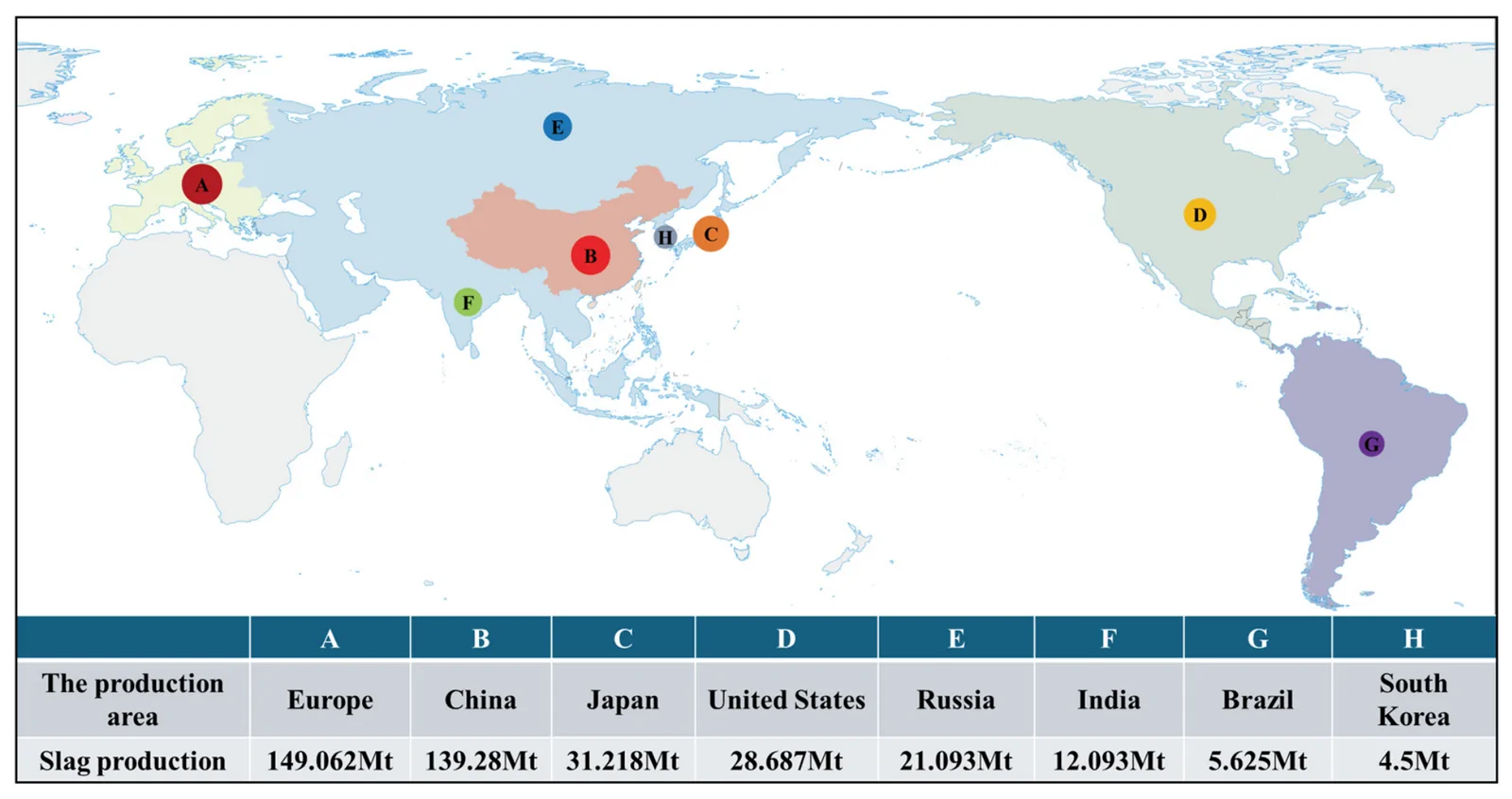
Spatial distribution map of steel slag output across countries (redrawn with permission from Reference [26]; open access, Elsevier).

**Figure 2:**
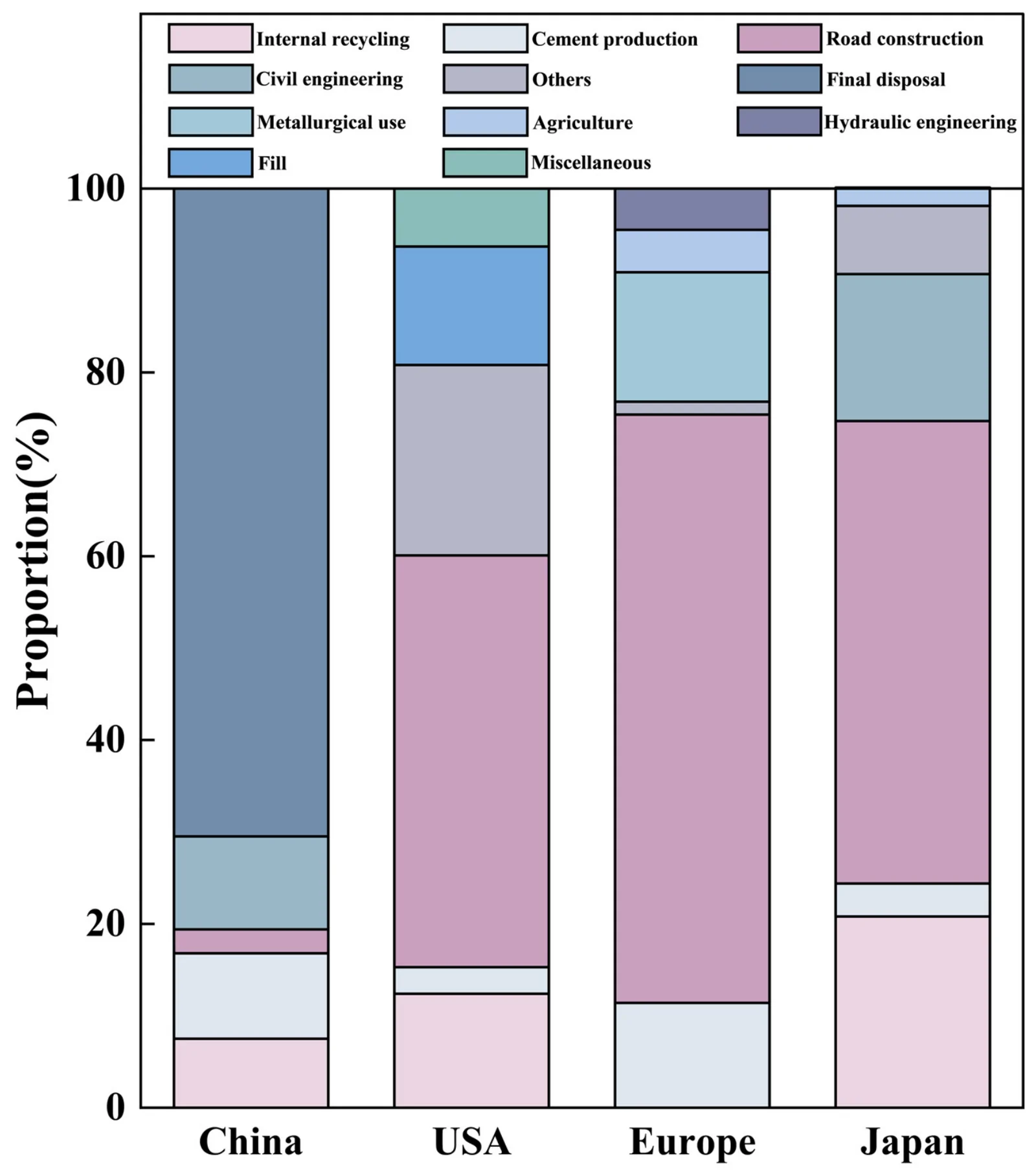
Proportions of steel slag utilization: (a) China; (b) USA; (c) Europe; (d) Japan (the data were derived from Reference [31]; open access).

**Figure 3:**
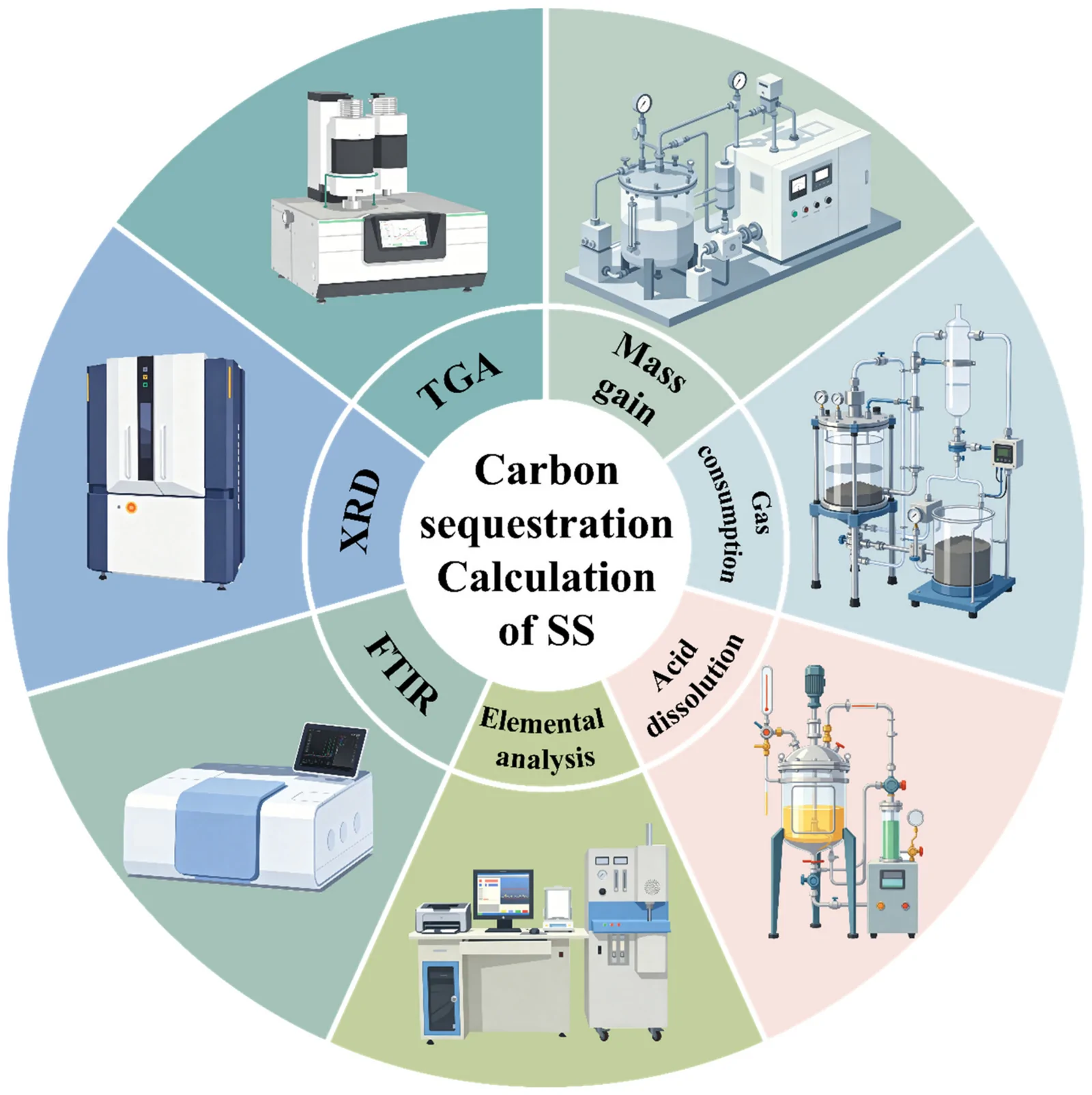
Mainstream quantification approaches for CO_2_ sequestration capacity of steel slag. (The three isometric experimental setup schematics in this figure were generated by Doubao 1.6 with separate prompts for each subplot: For the Mass gain section: “Generate an apparatus diagram for measuring the carbon sequestration capacity of steel slag via the mass gain method.” For the Gas consumption section: “Generate an apparatus diagram for measuring the carbon sequestration capacity of steel slag via the gas consumption method.” For the Acid dissolution section: “Generate an apparatus diagram for measuring the carbon sequestration capacity of steel slag via the acid dissolution method”).

**Figure 4:**
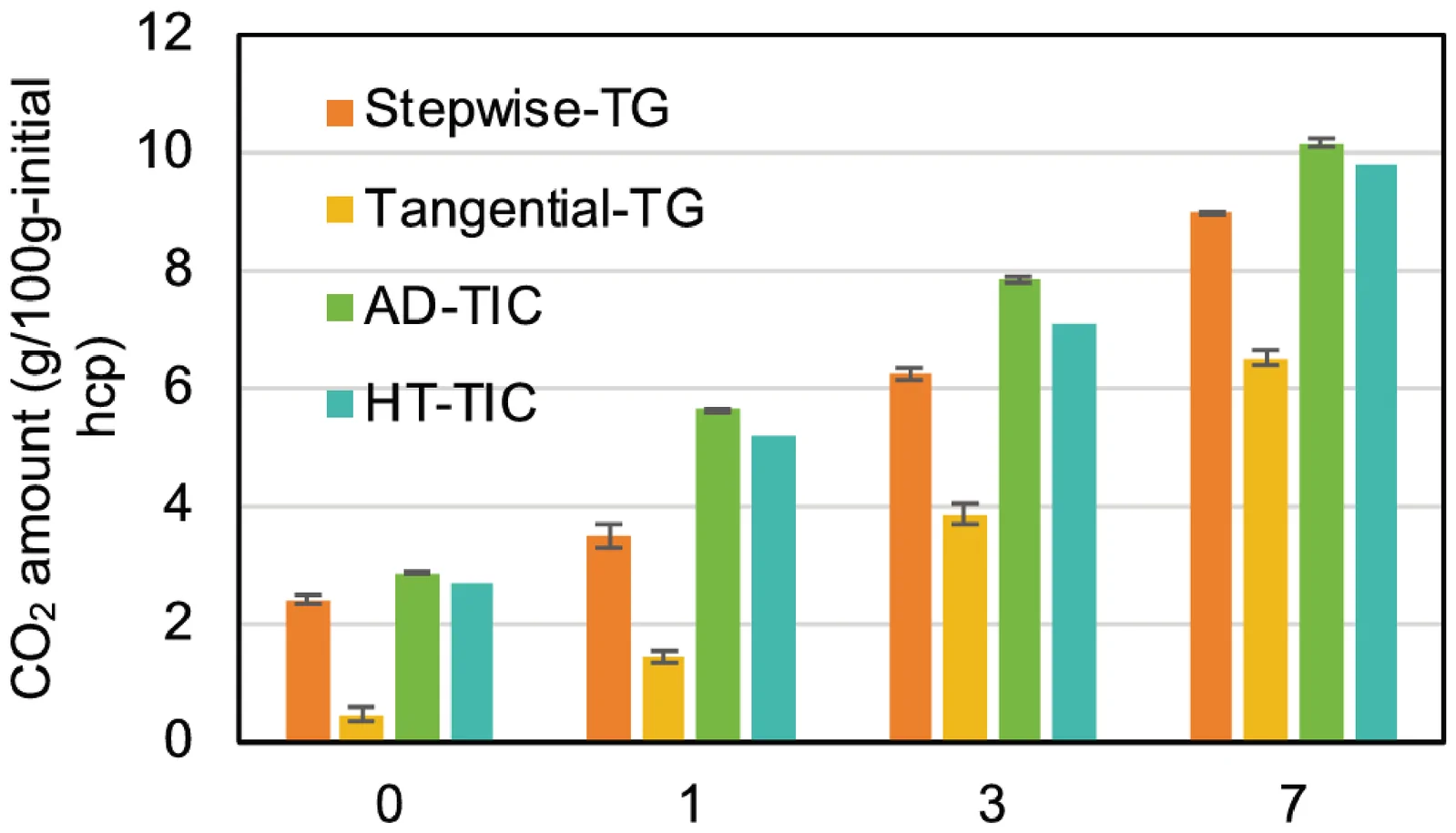
Discrepancies in CO_2_ sequestration quantification calculated by four analytical methods (data in this figure are derived from Reference [42], open access, Elsevier; error bars represent standard deviation (SD), with sample size *n* ≥ 3).

**Figure 5:**
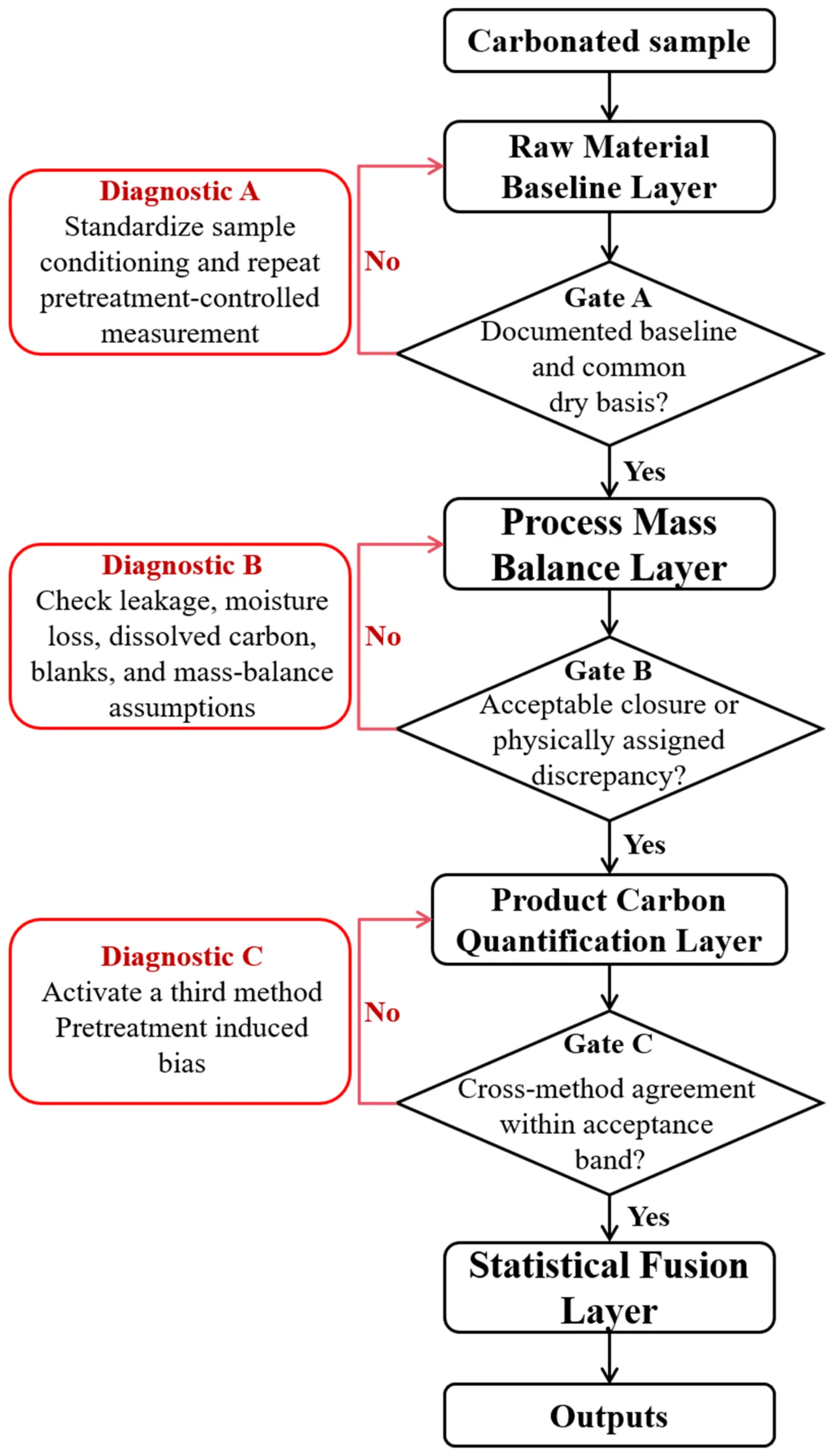
Decision framework of the four-layer evidence chain for quantification of steel slag CO_2_ sequestration.

**Table 2:** Operational carbon pools, observables, and their correspondence to the seven core quantification techniques.

Quantity	Operational Definition	Primary Technique(s)	Quantitative Interpretation
*C*_0_	Native/background inorganic carbon present before intentional carbonation	TIC/elemental analysis; TGA; acid digestion; XRD	Must be measured on a batch- and pretreatment-matched raw-slag baseline and subtracted from final solid carbon.
*G*	Corrected gas-phase CO_2_ disappearance during reaction	Gas consumption method	*G* = *C_s_*_,_*_new_* + *C_l_* + *C_p_* + *ε_sys_*; therefore *G* is an apparent process uptake unless liquid, trapped-gas, leakage, and sensor terms are resolved.
*Cl*	Dissolved inorganic carbon in liquid, condensate, or washings	Liquid-phase TIC/IC/alkalinity as auxiliary closure measurements	Not permanent solid sequestration at the sampling instant unless subsequently precipitated; required for gas–liquid–solid mass closure.
*C_p_*	Physically retained, adsorbed, or pore entrapped CO_2_	Desorption/degassing checks; process balance	A transient pool that can inflate gas-consumption estimates but should not be counted as stable mineralized carbon.
*C_s,new_*	Newly formed stable solid inorganic carbon retained after carbonation	Elemental TIC; acid digestion; TGA; combustion-infrared after TOC/background correction	Primary endpoint for net sequestration: *C_s_*_,_*_new_* = *C_solid_*_,_*_final_* *− C*_0_ on a consistent dry-mass basis.
*C_x_*	Crystalline carbonate carbon	XRD-Rietveld	*C_x_* ≤ *C_s_*_,_*_new_*. A *C_s_*_,_*_new_* *− C_x_* gap indicates amorphous/poorly crystalline or other XRD-invisible inorganic carbon.
*M*	Dry solid mass change before/after carbonation	Mass gain method	*M* = *C_s_*_,_*_new_* + non-carbon mass changes. Hydration, salts, leaching, and particle loss must be corrected before *M* can approximate *C_s_*_,_*_new_*.

**Table 3:** Origins, manifestations, and correction strategies of quantification discrepancies among analytical methods.

Source of Discrepancy	Mechanism and Intrinsic Principle	Typical Phenomena	Prior Inspection Methods	Correction Strategies	Intrinsic Error Category
Unsubtracted native carbonates in raw slag	The gas-phase cumulative CO_2_ consumption records carbonate formation over the full reaction lifecycle; an inconsistent definition of metrological baseline leads to mixing of historical background carbon and newly sequestered CO_2_	Overestimated CO_2_ sequestration capacity for all solid-phase endpoints; severe mass imbalance between gas-phase carbon and solid-phase mass increment	Blank test with batch-matched raw slag, TIC, TGA, XRD, phase characterization	Background subtraction using blank raw slag from the identical batch	Systematic bias from inconsistent statistical accounting criteria
Inconsistent moisture status	Hydration mass gain and carbonation mass gain share identical physical origins; mass metrology baselines differ between wet and dry samples, lacking unified conversion standards for wet/dry substrates	CO_2_ sequestration results from the mass gain method are remarkably higher than the TGA test data	Moisture content measurement, blank CO_2_ water vapor test	Unified drying procedure for all samples; separate deduction of hydration-induced mass increment	Inconsistent metrological reference baseline
Presence of amorphous carbonates	XRD only identifies long-range ordered crystalline phases; acid digestion captures both crystalline and amorphous carbonate fractions; the two methods differ in detection thresholds and fundamental principles	TIC-measured values are significantly higher than XRD-quantified carbon content	FTIR characterization of amorphous carbonates, joint quantification via XRD and TGA	Report amorphous phase fractions and reduce the weighting factor of the XRD quantification results	Detection limit deviation caused by the material crystallinity difference
Unaccounted DIC in liquid phase	Reversible dissolution equilibrium exists in the gas–liquid CO_2_ system; partial CO_2_ remains in the liquid as ionic species; only solid-phase carbon is counted, breaking the three-phase mass balance	Gas-phase consumed CO_2_ is higher than the measured carbon content of solid products	Liquid-phase TIC, pH, and alkalinity testing	Establish a gas–liquid–solid three-phase carbon balance equation	Incomplete coverage of the CO_2_ sequestration accounting boundary
Incomplete acid digestion	Carbonates are physically encapsulated by gel-like substances; diffusion is restricted under low-temperature acidolysis, while high-temperature thermal digestion eliminates encapsulation barriers	TIC test results are lower than data from TGA and elemental combustion analysis	Particle size gradient test, acid digestion duration gradient test, calcium carbonate matrix spike recovery test	Ultra-fine grinding, extended acidolysis duration, matrix spike recovery calibration	Dynamic limitation from microscale mineral encapsulation
Residual organic carbon/additive interference	Low-temperature acidolysis only decomposes inorganic carbon; high-temperature combustion oxidizes organic and inorganic carbon simultaneously; elemental analysis cannot automatically separate carbon species	Measured carbon from elemental TC/TOC deviates largely from TIC values	Sequential TOC-TIC testing, blank test with pure additives	Stepwise subtraction of organic carbon background signals	Inseparable carbon species morphology
Gas circuit leakage or sensor drift	Dynamic temperature-pressure fluctuations under dry conditions, pipeline leakage, and sensor temperature drift distort gas-phase concentration and volume signals	Abnormal carbon consumption curves; excessive closure error of the overall carbon mass balance	Air tightness test, blank calibration with inert reference materials	Pre-experiment leakage inspection; real-time gas-phase correction under constant temperature and pressure throughout the test	Instrument drift of the dynamic monitoring system

**Table 4:** Standardized workflow for multi-method collaborative quantification of CO_2_ sequestration in steel slag.

Stage	Objective	Recommended Methods	Key Outputs	Criterion/Threshold Requirement
Raw Material Baseline Layer	Establish a baseline zero point for CO_2_ sequestration capacity	TIC/TC/TOC, LOI, XRF, XRD, moisture content measurement	Raw sample TIC, loss on ignition, theoretical CO_2_ sequestration potential	Raw TIC and LOI test reports are mandatory
Process Mass Balance Layer	Record gas-phase CO_2_ consumption or solid-phase mass increment	Gas consumption method, mass gain method	Cumulative gas consumption, kinetic rate curves, and CO_2_ utilization efficiency	Blank reactor and inert reference sample leakage correction required
Product Carbon Quantification Layer	Quantify newly generated stable inorganic carbon	At least two methods among TGA, acid digestion, elemental analysis, and combustion-infrared spectroscopy	Raw sample CO_2_ content, baseline-subtracted net CO_2_ increment	Relative deviation between two independent methods < 10% as a minimum mutual verification standard
Statistical Fusion Layer	Generate auditable quantitative results	Weighted averaging, uncertainty propagation	X ± U (k = 2)	Report repeatability, calibration, sampling and model component uncertainties

## Data Availability

No new data were created or analyzed in this study. Data sharing is not applicable to this article.
